# Supplementary material for: Chronological Incongruences between Mitochondrial and Nuclear Phylogenies of Aedes Mosquitoes
Source: Life (Basel). 2021 Feb 25;11(3):181. doi: 10.3390/life11030181 (PMC7996624; doi:10.3390/life11030181)

Supplementary figure S1: Maximum Liklehood analyses using RAXML and GTR+G

a. nuclear

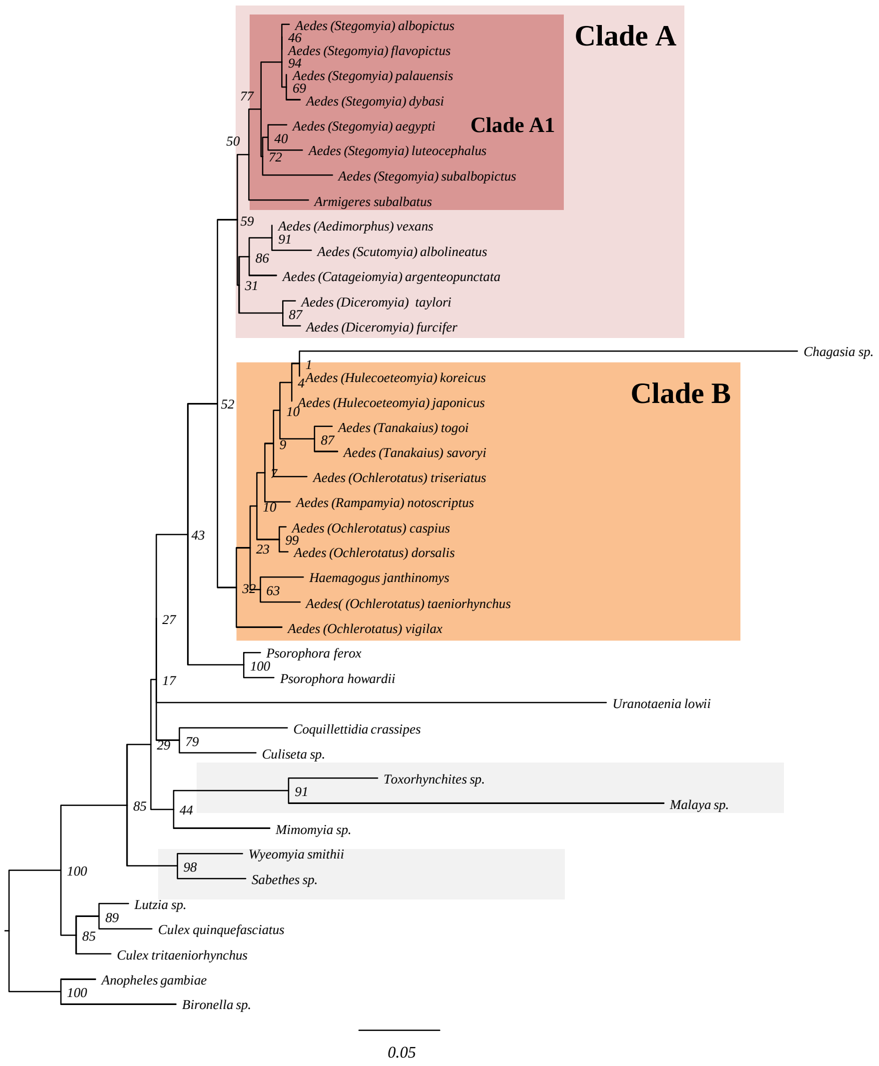

b. mitochondrial

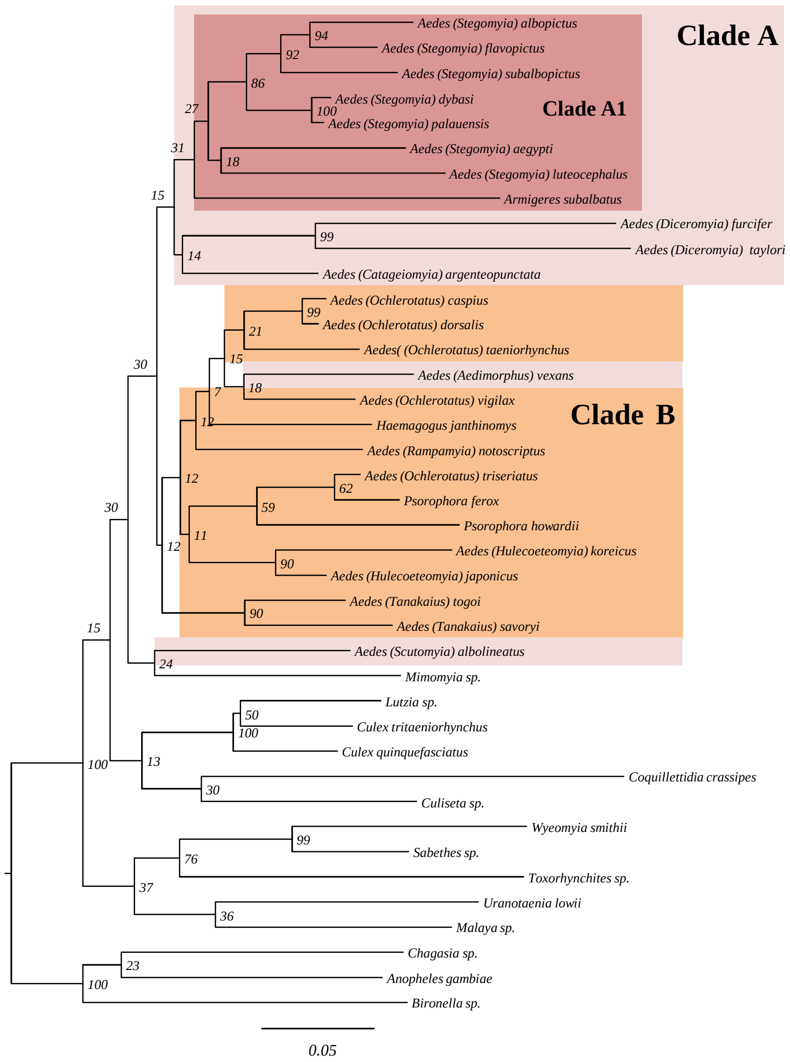

Supplementary figure S2: Bayesian trees of concatenated datasets

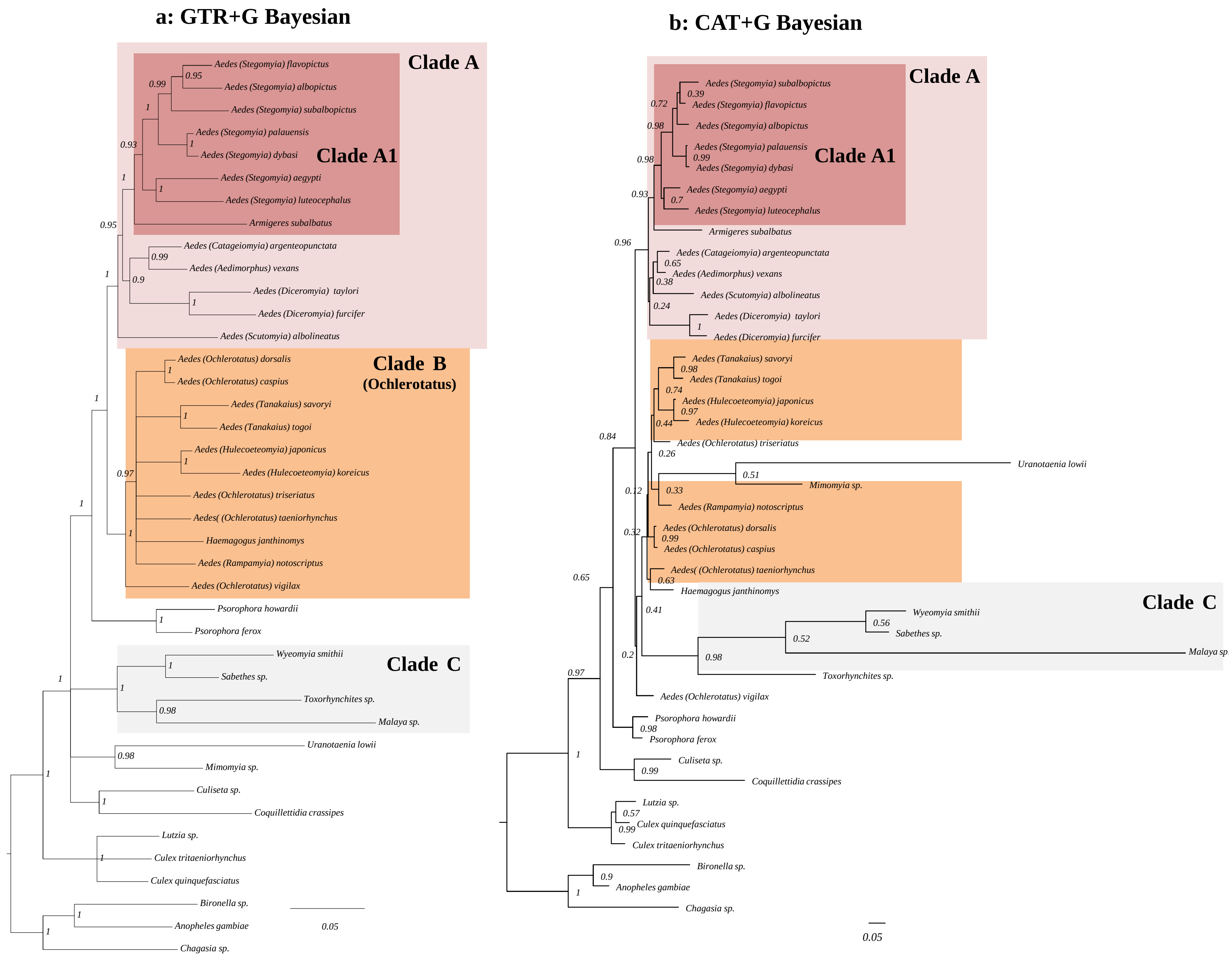

Supplementary figure S3: concatenated dataset, full trees

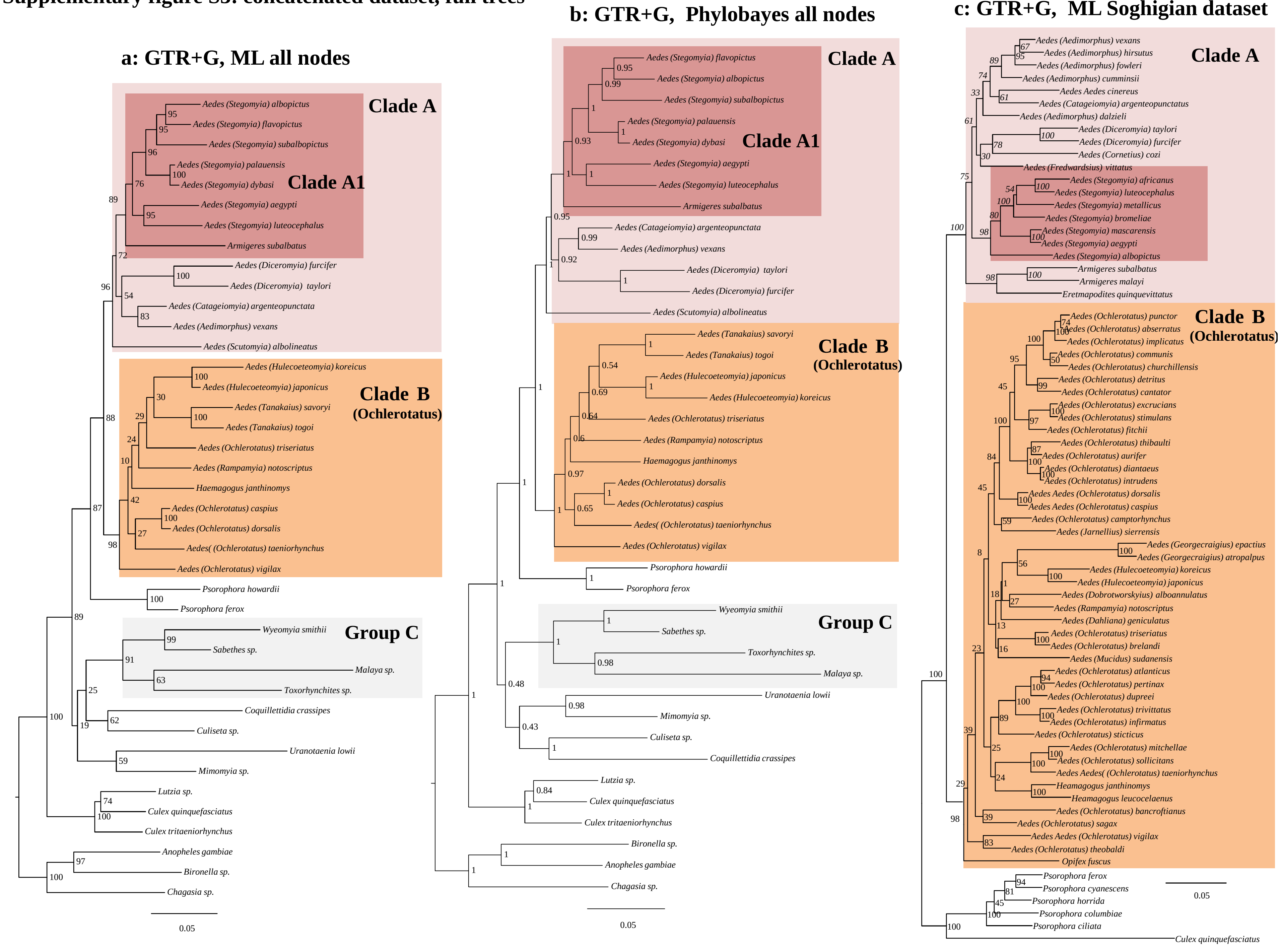

Supplementary figure S4: Soghigian dataset CAT+G full trees

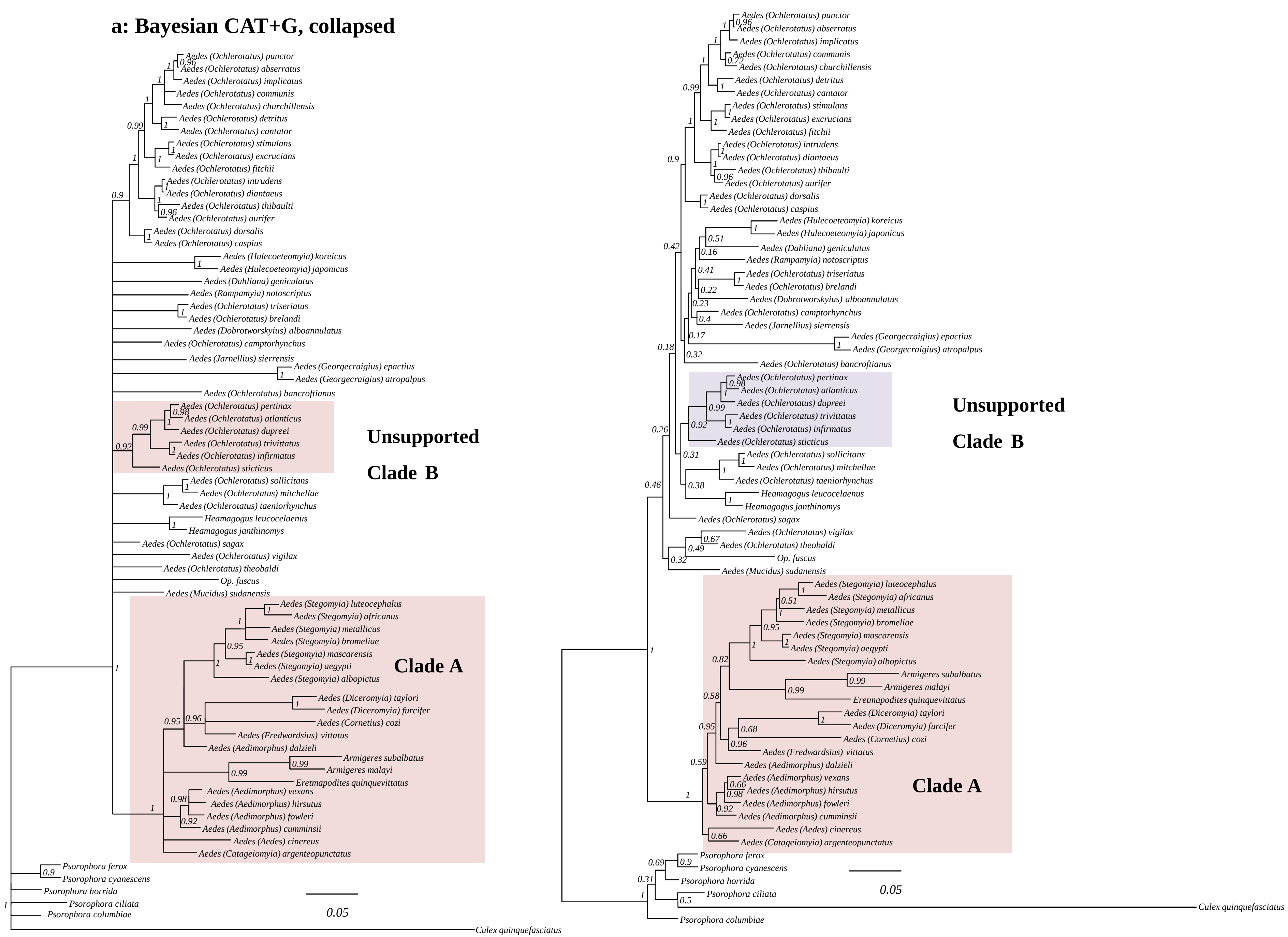

Supplementary figure S5: Cleaned datasetsBEAST trees.

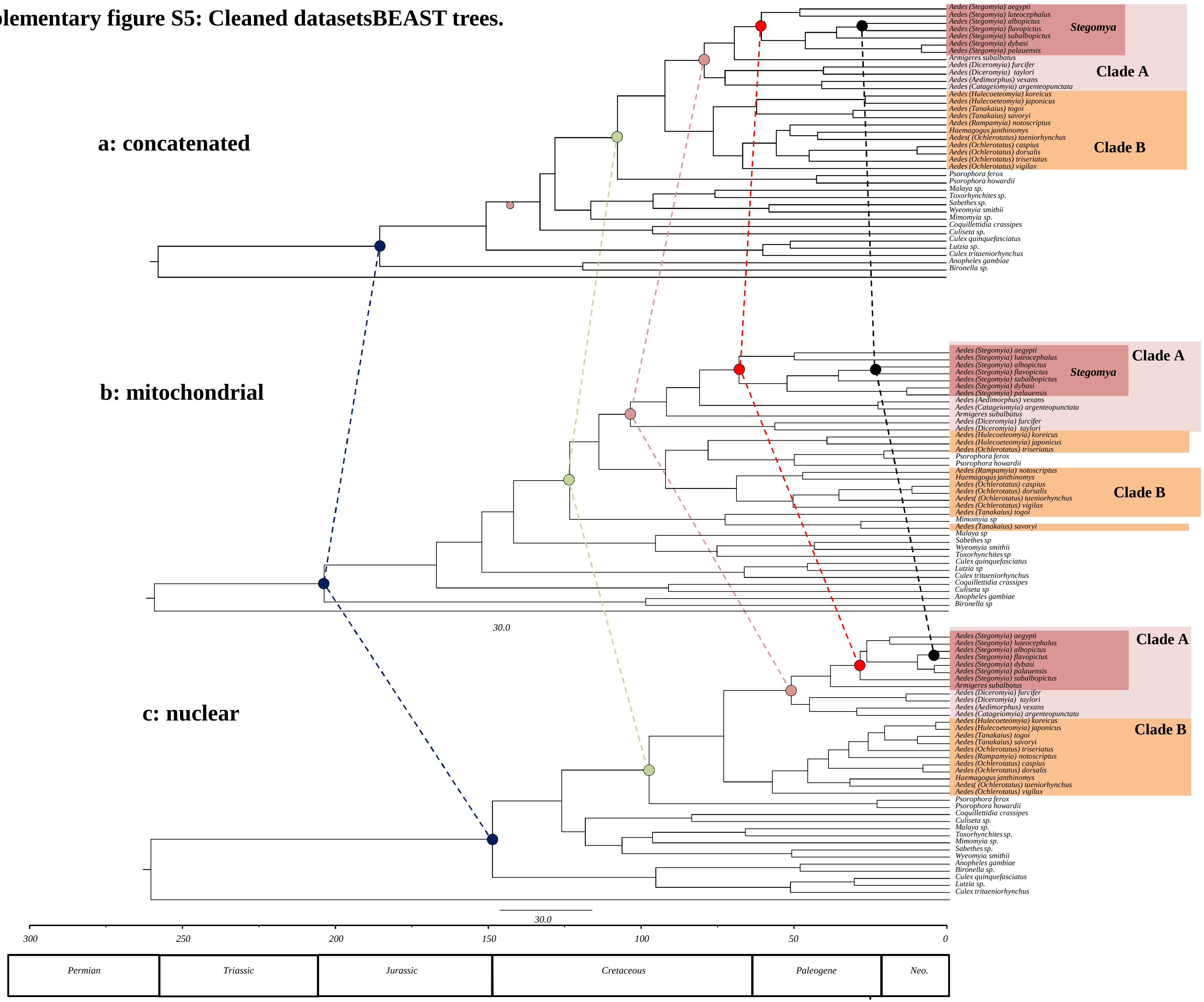

Supplementary figure S6: Cleaned datasets BEAST trees. Posterior mean rates at nodes

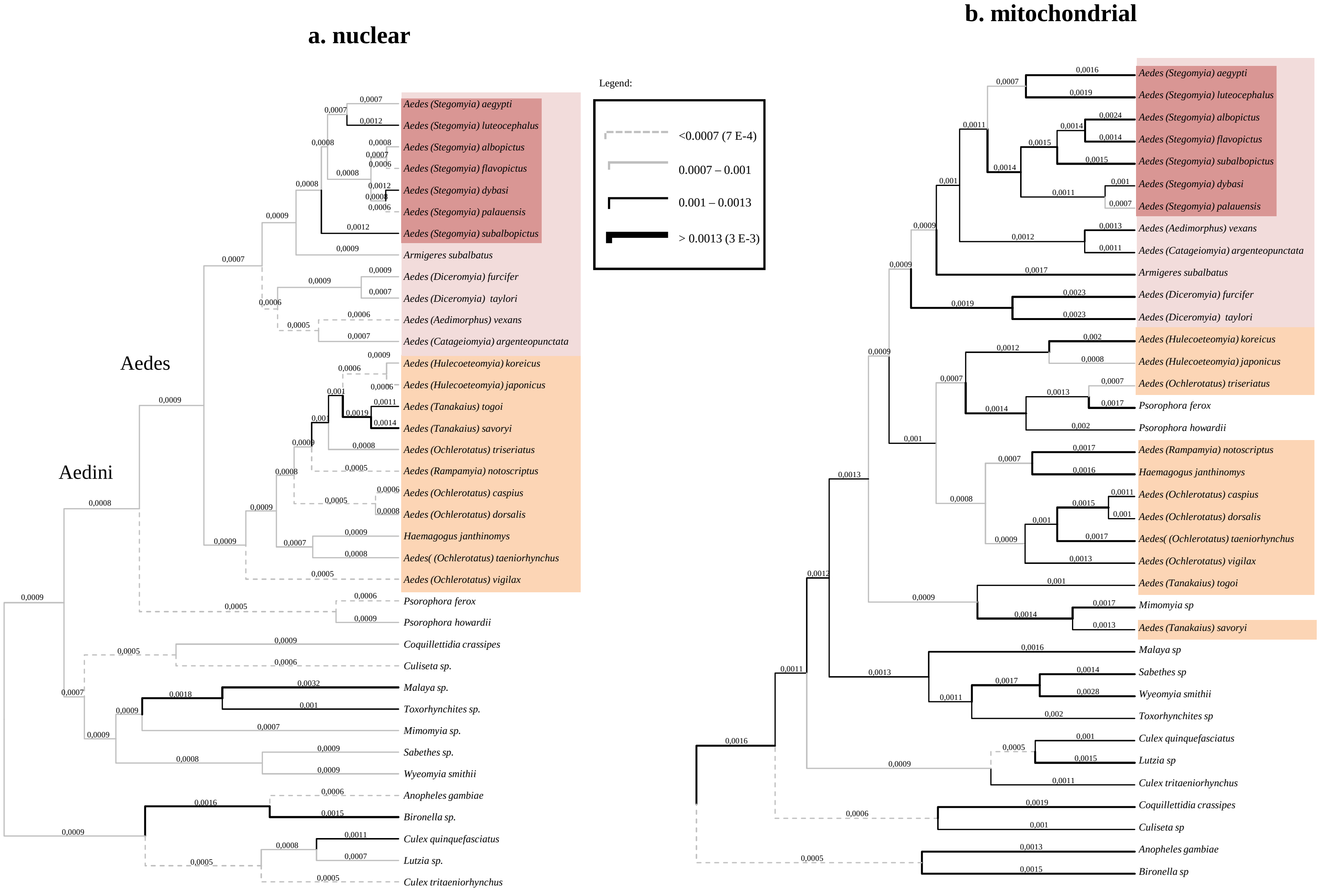

Supplementary figure S7: Partitioned Soghigian BEAST trees.

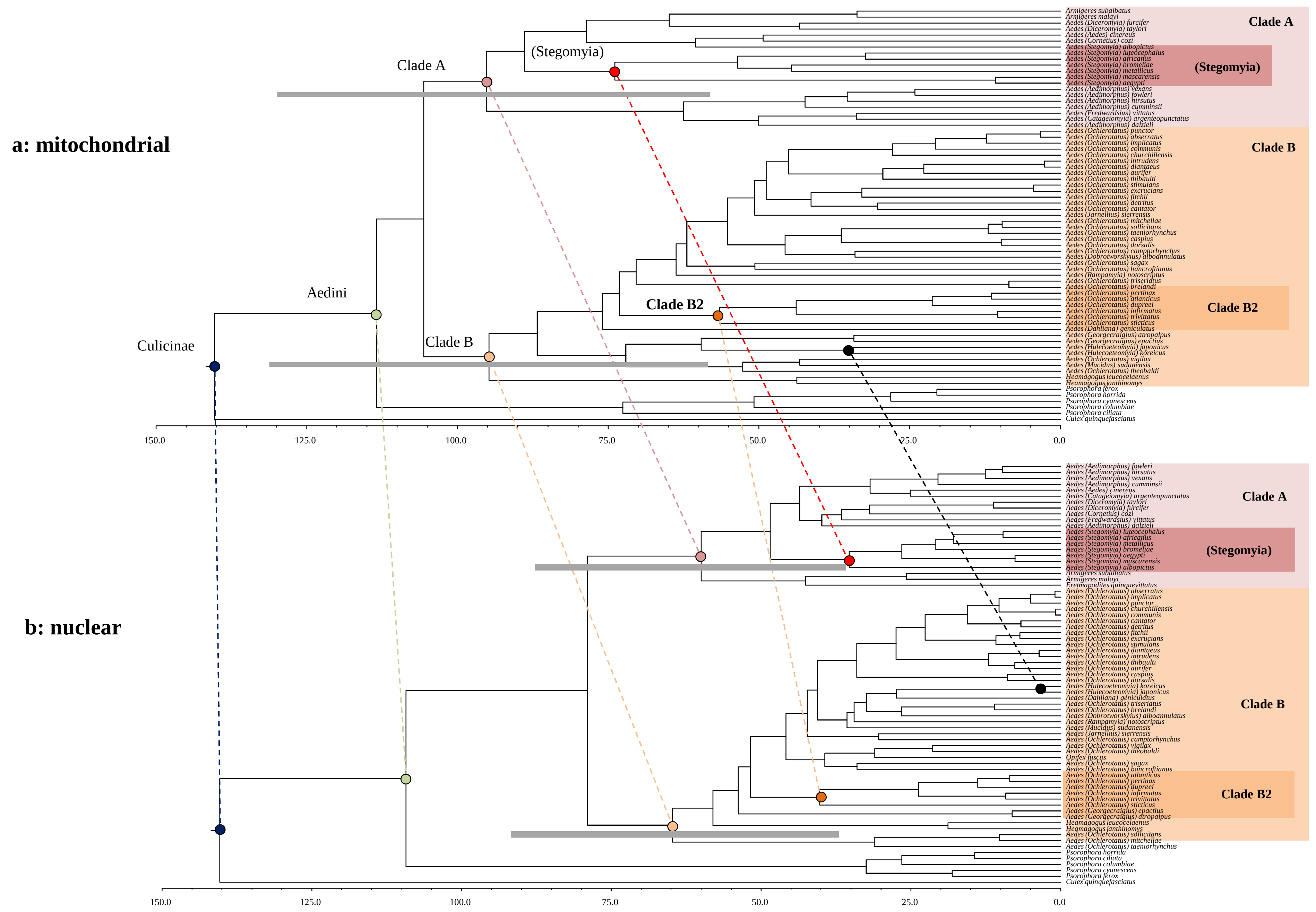

Supplementary figure S8: Phylobayes timetrees using the CAT+G model

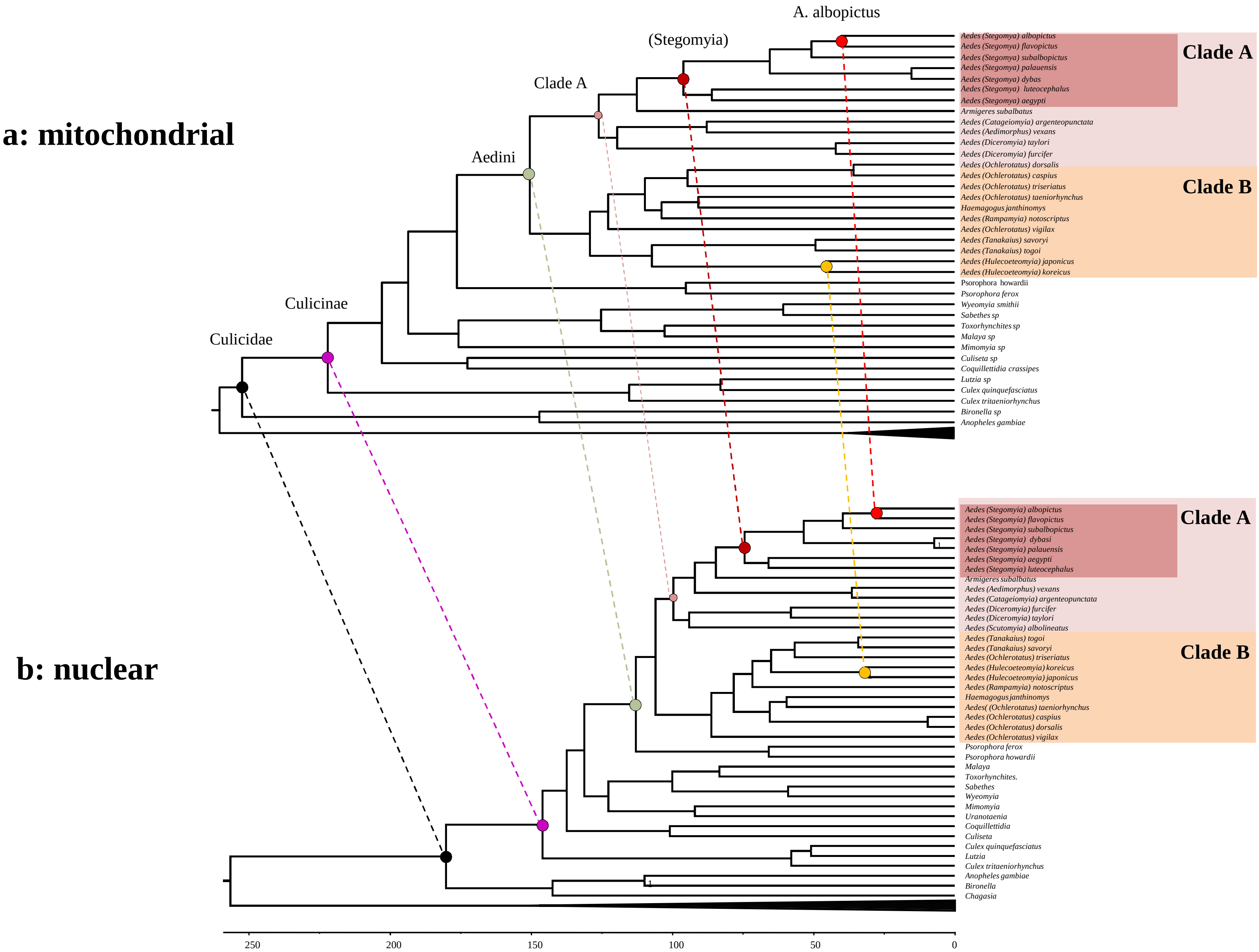

Supplement: Supplementary file 1 [file life-11-00181-s001.zip › Zadra_Life_Suppl.FiguresS1-S8.pdf]
