# Supplementary material for: Chronological Incongruences between Mitochondrial and Nuclear Phylogenies of Aedes Mosquitoes
Source: Life (Basel). 2021 Feb 25;11(3):181. doi: 10.3390/life11030181 (PMC7996624; doi:10.3390/life11030181)
Supplement: Supplementary file 1 [file life-11-00181-s001.zip › Zadra_Life_Suppl.Table S1.pdf]

Table S1. Species names and gene list

Species name	Nuclear genes				Mitochondrial genes			
	**28S**	**18S**	**Enolase**	**Arg-kinase**	**16S**	**CoxI**	**CoxII**	**NAD4**
Aedes (Stegomya) aegypti	MG242540.1	MG232525.1	MG232460.1	MG232457.1	EU352212.1			
Aedes (Scutomya) albolineatus	KF687500.1					KF564650.1	KC913592.1	
Aedes (Stegomya) albopictus	MG242543.1	X57172.1	MG232463.1	MG232454.1	AY072044.1			
Aedes (Stegomya) dybasi	DQ397943.1				DQ397925.1	DQ397913.1		
Aedes(Stegomya) flavopictus	DQ397936.1				DQ397929.1	DQ397907.1		
Aedes (Diceromyia) furcifer	MG242572.1	MG232557.1	MG232487.1	MG232431.1		KU187185.1	AY645287.1	
Aedes (Huleocoetomya) koreicus	GU229913.1					KT962063.1	GU229897.1	KT945239
Aedes (Stegomya) luteocephalus	MG242581.1	MG232566.1	MG232495.1	MG232424.1		AY645234.1	AY645300.1	JX427527.1
Aedes (Rampamyla)notoscriptus	MG242585.1	MG232570.1	MG232499.1	MG232420.1	KM676218.1			
Aedes (Stegomya) palauensis	DQ397942.1				DQ397924.1	DQ397896.1		
Aedes (Stegomya) subalbopictus	KF687510.1					EU259301.1	KC913583.1	
Aedes (Diceromyia) taylori	MG242596.1	MG232580.1	MG232508.1	MG232411.1		AY645228.1	AY645295.1	
Aedes (Tanakayus) togoi	GU229910.1		LC026054.1			LC025711.1	GU229895.1	GU229922.1
Aedes (Aedimorphus) vexans	MG242602.1	AM071382.1	MG232513.1	MG232406.1		AY645243.1	AY645304.1	
Anopheles gambiae	GCA_000005575.1 (genome)				MG753708.1			
Armigeres subalbatus	MG242607.1	MG232591.1	MG232516.1	MG232402.1	AY439688.1	KM497419.1	HQ398972.1	AY440746.1
Bironella_sp.		AF417796.1	GQ906881.1	GQ906808.1	MF381612.1			
Calliphora vomitoria	MF281681.1	MF992252.1			KT444440.1			
Aedes (Catageiomyia) argenteopunctata	MG242545.1	MG232530.1	MG232464.1	MG232453.1		MG242468.1		
Chagasia_sp.	AF417831.1	AF417797.1			MF381717.1			
Chrysomya megacephala	KY197787.1	FJ025483.1			KT272787.1			
Coquillettidia crassipes	KF687592.1	AY988454.1	GQ906883.1			KF564770.1	KF687365.1	
Culex quinquefasciatus	XM_001865926.1	XM_001849752.1	XM_001842566.1	XM_001849602.1	GU188856.2			
Culextritaeniorhynchus	KF687515.1	U48385.1			KT852976.1			
Culiseta sp.	KF687543.1	AY988451.1	GQ906882.1 fla	GQ906809.1		AF425848.1		
Drosophila pseudoobscura	HQ110539.1	XR_053284.1	AF025805.1	XM_015187032.1	FJ899745.1			
Drosophila sechellia		XR_048770.1	XM_002041688.1		AF200832.1			
Drosophila simulans	HQ110541.1	AY037174.1	DQ864222.1	XM_016184539.1	AY518672.1			
Drosophila yakuba	HQ110545.1		DQ864189.1	XM_002093328.2	KF824886.1			
Haemagogus janthinomys	MG242608.1	MG232592.1	MG232517.1	MG232401.1	KT372555.1			

Lucilia caesar	MF694304.1	FJ025492.1			KM657111.1			
Lucilia cuprina	FR719299.1			JQ088101.1	JX913750.1			
Lucilia sericata	KY197804.1:	KR133393.1			KT272854.1			
Lutzia sp.	KF687516.1					HQ398896.1	HQ398958.1	
Malaya sp.	KF687541.1			GQ906814.1		EU259293.1	KF687373.1	
Mimomyia sp.	KF687538.1		GQ906889.1	GQ906816.1			KU380447.1	
Aedes (Ochlerotatus) caspius	MG242555.1	MG232540.1	MG232473.1	MG232444.1		KM452934.1		
Aedes (Ochlerotatus) dorsalis	MG242565.1		MG232480.1	MG232436.1	KU880657.1	KR691544.1	KC913569.1	
Aedes (Huleocoetomya) japonicus	MG242580.1	MG232565.1		MG232425.1	DQ397915.1	DQ397889.1 KC913579.1		DQ470164.1
Aedes (Ochlerotatus) taeniorhynchus	MG242595.1	MG232579.1	MG232507.1	MG232412.1		KT766538.1	HQ853680.1	
Aedes (Ochlerotatus) triseriatus	MG242600.1	MG232584.1	MG232511.1	MG232408.1		AF417730.1	AF417766.1	
Aedes (Ochlerotatus) vigilax	MG242603.1	MG232587.1	MG232514.1	MG232405.1	MK575484.1			
Psorophora ferox	MG242613.1	MG242613.1	MG232521.1	MG232396.1		MF172349.1		
Psorophora howardii	MG232599.1	MG232599.1	MG232523.1			MG242538.1		
Sabethes sp.				GQ906821.1	NC_037498.1			
Sarcophaga sp.	KU746469.1	AF322419.1			KM881633.1			
Aedes (Tanakaius) savoryi	LC025745.1		LC026055.1			LC025656.1		
Toxorhynchites sp.	KC177664.1	AY988455.1	GQ906895.1	GQ906823.1		AF425849.1		
Uranotaenia lowii	AF417833.1	AF417799.1		GQ906826.1		AF417728.1	AF417764.1	
Wyeomyia smithii			GQ906899.1	GQ906827.1		HM136816.1		
